# CRNDE affects the malignant biological characteristics of human glioma stem cells by negatively regulating miR-186

**DOI:** 10.18632/oncotarget.4509

**Published:** 2015-07-13

**Authors:** Jian Zheng, Xiao-dong Li, Ping Wang, Xiao-bai Liu, Yi-xue Xue, Yi Hu, Zhen Li, Zhi-qing Li, Zhen-hua Wang, Yun-hui Liu

**Affiliations:** ^1^ Department of Neurosurgery, Shengjing Hospital of China Medical University, Shenyang 110004, China; ^2^ Department of Neurobiology, College of Basic Medicine, China Medical University, Shenyang 110001, China; ^3^ Institute of Pathology and Pathophysiology, China Medical University, Shenyang 110001, China; ^4^ Department of Physiology, College of Basic Medicine, China Medical University, Shenyang 110001, China

**Keywords:** long non-coding RNAs, CRNDE, microRNAs, miR-186, glioma stem cells

## Abstract

The long non-coding RNA Colorectal neoplasia differentially expressed (CRNDE) is a novel gene that activated early in colorectal neoplasia, but it is also up-regulated in many other solid tumors. Herein, the function and underlying mechanism of CRNDE in regulating glioma stem cells (GSCs) were investigated. We found that CRNDE expression was up-regulated while miR-186 expression was down-regulated in GSCs. Overexpression of CRNDE could promote the cellular proliferation, migration, invasion and inhibit the apoptosis in GSCs. Overexpression of miR-186 exerted functions of inhibiting the proliferation, migration and invasion of GSCs and promoting apoptosis. And CRNDE decreased the expression levels of XIAP and PAK7 by binding to miR-186 and negatively regulating it. In addition, miR-186 binded to XIAP and PAK7 3′UTR region, and decrease the expression of them, thus regulating the expression levels of downstream target proteins such as caspase 3, BAD, cyclin D1 and MARK2. The *in vivo* effect of CRNDE and miR-186 showed that the tumor formation rate was minimum in tumor-bearing nude mice with the knockdown of CRNDE and the overexpression of miR-186. In conclusion, CRNDE played an oncogenic role of GSCs through the negative regulation of miR-186. Both CRNDE and miR-186 could be regarded as potential targets in the glioma therapy.

## INTRODUCTION

Glioma is the most prevalent primary malignant tumor in the adult human central nervous system (CNS), which is characterized by difficulty in early diagnosis and extremely poor prognosis. It is prone to metastasize and recur even after surgery, radiotherapy and chemotherapy. Patients with glioblastoma account for 50% of new cases of malignant CNS tumors every year, and the median survival period is 0.5∼1 year [1]. Glioma stem cells (GSCs) are a subgroup of glioma cells with the potentials of self-renewal, angiogenesis promotion and multi-differentiation. They are highly involved in the growth, metastasis, invasion and recurrence of glioma [2]. Therefore, it's necessary to find an effective way to control GCSs in the genetic therapy against gliomas.

Long non-coding RNAs (lncRNAs) possess various cellular biological behavior. For instance, lncRNAs regulate the expression of target genes by competitively binding to them and negatively regulating the activity and expression of microRNAs(miRNAs) [3]. LncRNAs also act as endogenous miRNA sponges to regulate the malignant biological behavior of tumor cells [4]. Colorectal neoplasia differentially expressed (CRNDE) was initially identified as an LncRNA in colorectal cancer and was found to be located on chromosome 16, which is of important significance in the proliferation, migration and invasion of colorectal tumor cells [5]. In addition, CRNDE is also overexpressed in a variety of other tumor cells such as melanoma and lymphocytic leukemia cells [6]. In the central nervous system, CRNDE has a specific expression pattern and contributes to neuronal differentiation; CRNDE is also the most highly expressed lncRNA in glioma, suggesting that CRNDE may be involved in the development and biological behavior of glioma [7].

MiRNAs are a group of non-coding RNAs with different expression levels in a variety of tumor cells. Accumulated evidence has shown that deregulation of miRNAs expression is involved in tumor formation and plays an important role in the regulation of tumor cells by inhibiting the translation of its downstream target genes or reducing the mRNA levels [8]. Previous studies have shown that miR-186 acted as a tumor suppressor and was down-regulated in many tumors such as lung adenocarcinoma, esophageal and colorectal cancers. In non-small cell lung carcinoma, miR-186 could inhibit the proliferation by inducing G(1)-S checkpoint arrest. In human colon cancer HCT116 cells, miR-186 also acted as a tumor suppressor to promote the cellular senescence through p53–p21 Cip1/WAF1 pathway [9–11]. However, the expression and function of miR-186 in gliomas still remain unclear.

X-linked inhibitor of apoptosis (XIAP) is a class of anti-apoptotic proteins and is highly expressed in hepatocellular carcinoma, pancreatic cancer and glioma [12, 13]. PAK7 (also known as PAK5), an evolutionarily conserved serine/threonine protein kinase, is highly expressed in glioma cells and promotes the cell proliferation, migration, invasion as well as inhibiting apoptosis [14, 15].

In this study, we aimed at investigating the expression levels of CRNDE and miR-186 in glioma stem cells and the effects of CRNDE on miR-186-induced regulation of XIAP and PAK7 as well as the underlying mechanism in the process.

## RESULTS

### CRNDE was highly expressed in GSCs while miR-186 was lowly expressed in glioma tissue and GSCs

**Figure 1:**
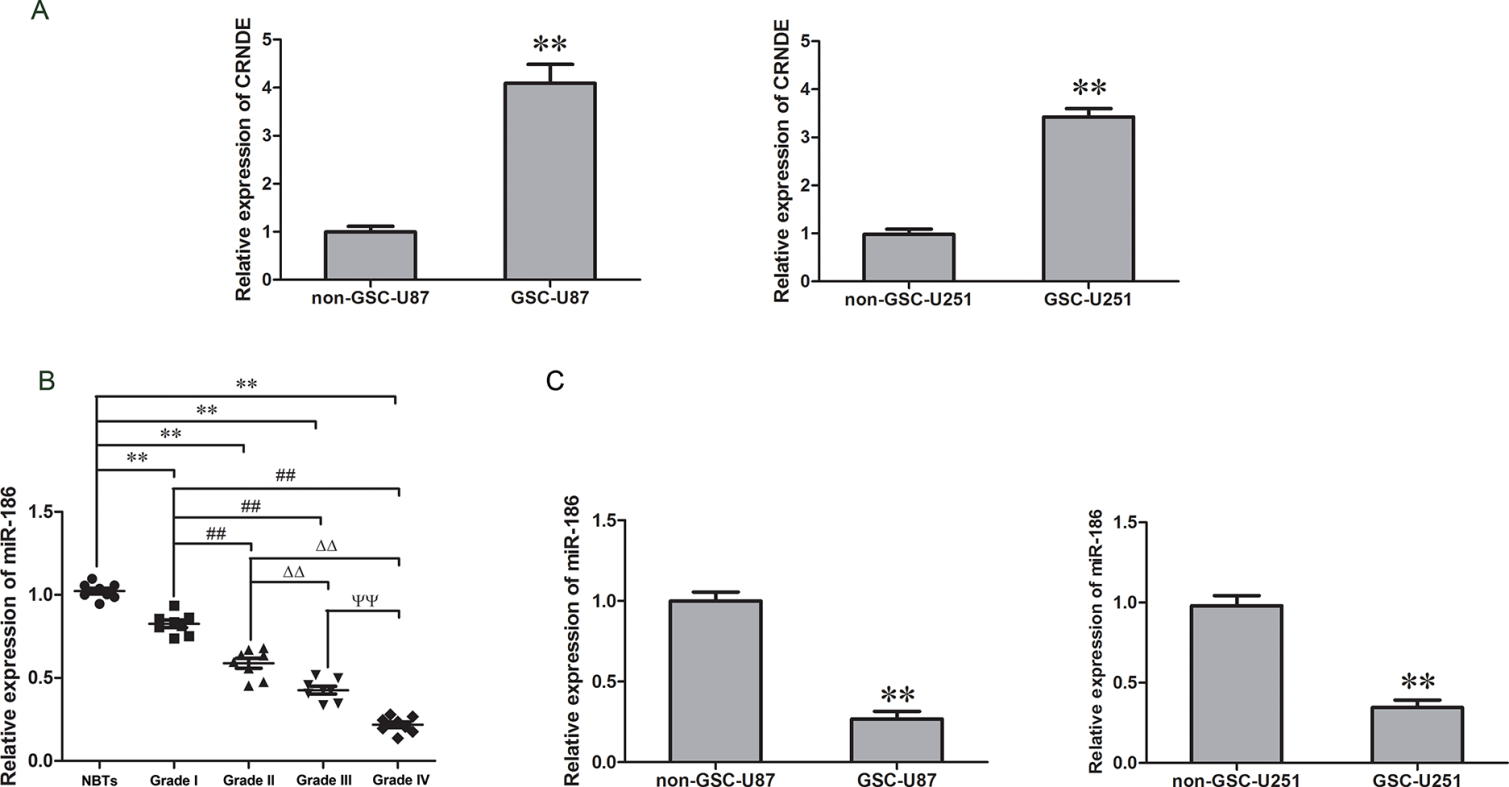
CRNDE expression in GSCs and miR-186 expression in glioma tissues and GSCs **A.** Expression levels of CRNDE in non-GSCs and GSCs. Data are presented as the mean ± SD (*n* = 5, each group). **P < 0.01 vs. non-GSCs group. **B.** Expression levels of miR-186 in glioma tissues of different grades and normal brain tissues (NBTs). Data are presented as the mean ± SD (*n* = 8, each group). **P < 0.01 vs. NBTs group; ^##^*P* < 0.01 vs. Grade I group; ^ΔΔ^*P* < 0.01 vs. Grade II group; ^ΨΨ^*P* < 0.01 vs. Grade III group. **C.** Relative expression of miR-186 in non-GSCs and GSCs. Data are presented as the mean ± SD (*n* = 5, each group). **P < 0.01 vs. non-GSCs group.

The expression levels of CRNDE and miR-186 in GSCs, non-stem cells (non-GSCs) and glioma tissues of different grades were detected with qRT-PCR. CRNDE expression levels in GSCs were increased compared with non-GSCs (Figure 1A). MiR-186 expression in glioma tissues was significantly decreased compared with the normal brain tissue (NBTs) and the expression was negatively correlated with the increasing pathological grades of glioma (Figure 1B). MiR-186 expression in GSCs was lower than that in non-GSCs (Figure 1C). These results implied that CRNDE served as an oncogene while miR-186 acted as a tumor suppressor gene and both of them would be involved in the biological processes of GSCs.

### Overexpression of CRNDE promoted the proliferation, migration and invasion and inhibited apoptosis in GSCs

**Figure 2:**
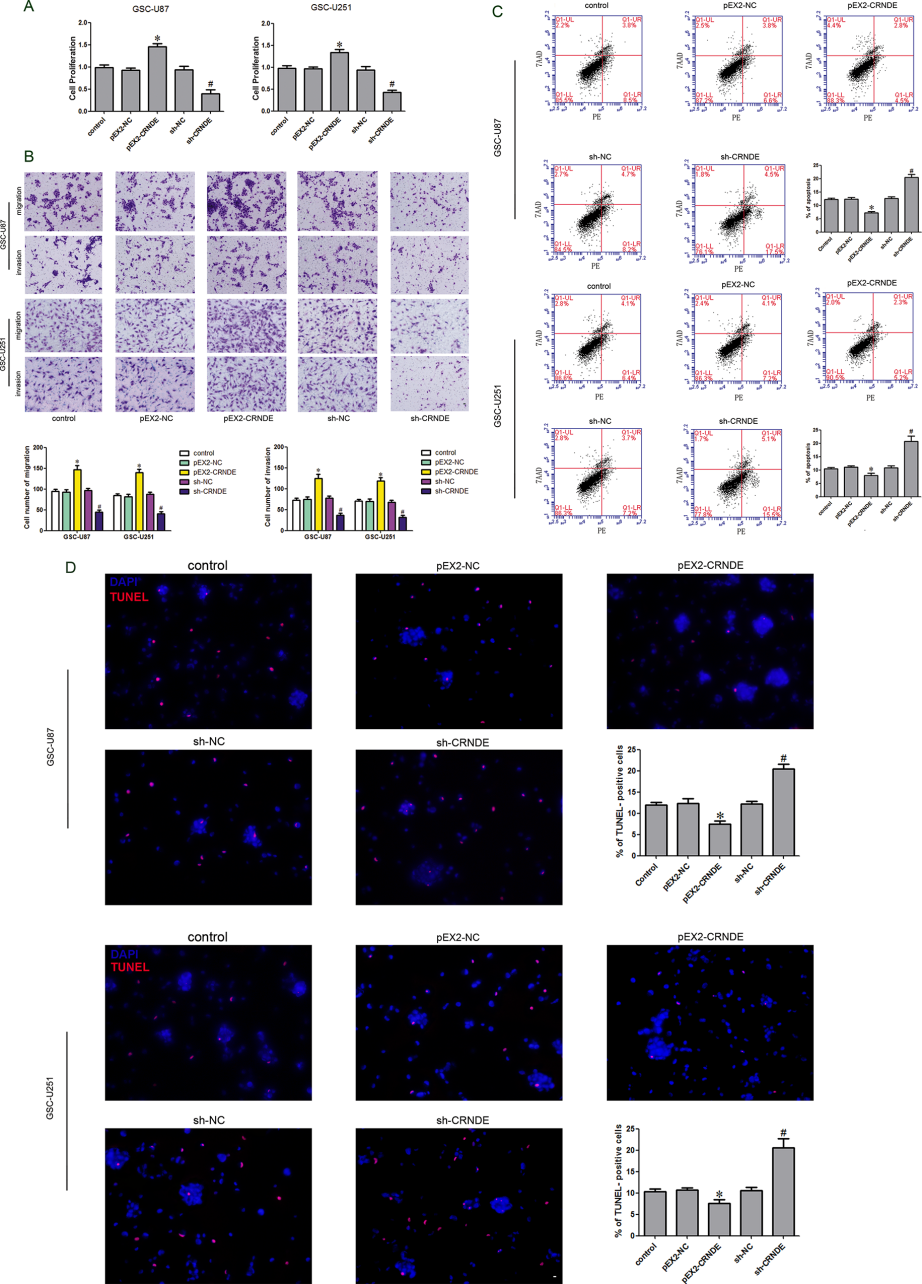
Effect of CRNDE on proliferation, apoptosis, migration and invasion of GSCs **A.** CCK-8 assay was applied to evaluate the proliferation effect of CRNDE on GSCs. **B.** Quantification of migration and invasion cells with different expression levels of CRNDE. Representative images and accompanying statistical plots were presented. **C.** Flow cytometry analysis of GSCs with the expression of CRNDE changed. **D.** Representative images of experiments of TUNEL assay (red) of GSCs with the expression of CRNDE changed. Data are presented as the mean ± SD (*n* = 5, each group). *P < 0.05 vs. pEX2-NC group; ^#^*P* < 0.05 vs. sh-NC group. Scale bars represent 20 μm.

After the CRNDE expression in GSCs was investigated, we further analyzed the effect of CRNDE on the proliferation, migration, invasion and apoptosis of GSC-U87 and GSC-U251 cells. The stably transfected stem cell lines with overexpression or knockdown of CRNDE were established and identified by CCK-8 assay. The proliferation rate was significantly higher in the pEX2-CRNDE group than that in the pEX2-NC group, and was obviously lower in the sh-CRNDE group than that in the sh-NC group (Figure 2A). Flow cytometry analysis was applied to detect the apoptosis and the results demonstrated that, the apoptotic rates in the pEX2-NC group and the sh-NC group were 10.4% ± 1.32% and 7.3% ± 0.97% in the GSCs with CRNDE overexpression and 20.6 ± 1.58% in the GSCs with CRNDE silencing groups separately (Figure 2C). TUNEL assay was used to further assess the apoptosis. Results showed that the TUNEL-positive cells (%) in the pEX2-CRNDE group was significantly decreased compared with the pEX2-NC group, whereas TUNEL-positive cells (%) in the sh-CRNDE group was significantly increased (Figure 2D). There was statistically significant difference among different groups. Migration and invasion assay results showed that the numbers of migrating and invading cells in the pEX2-CRNDE 2 groups were significantly increased compared with the control group, whereas migration and invasion were significantly attenuated in the sh-CRNDE 2 groups compared with the control group (Figure 2B). The above results indicated that CRNDE was expressed in the GSCs and affected the cellular proliferation, migration, invasion and apoptosis as an oncogene.

### Knockdown of miR-186 promoted the proliferation, migration and invasion of GSCs and inhibited the apoptosis

**Figure 3:**
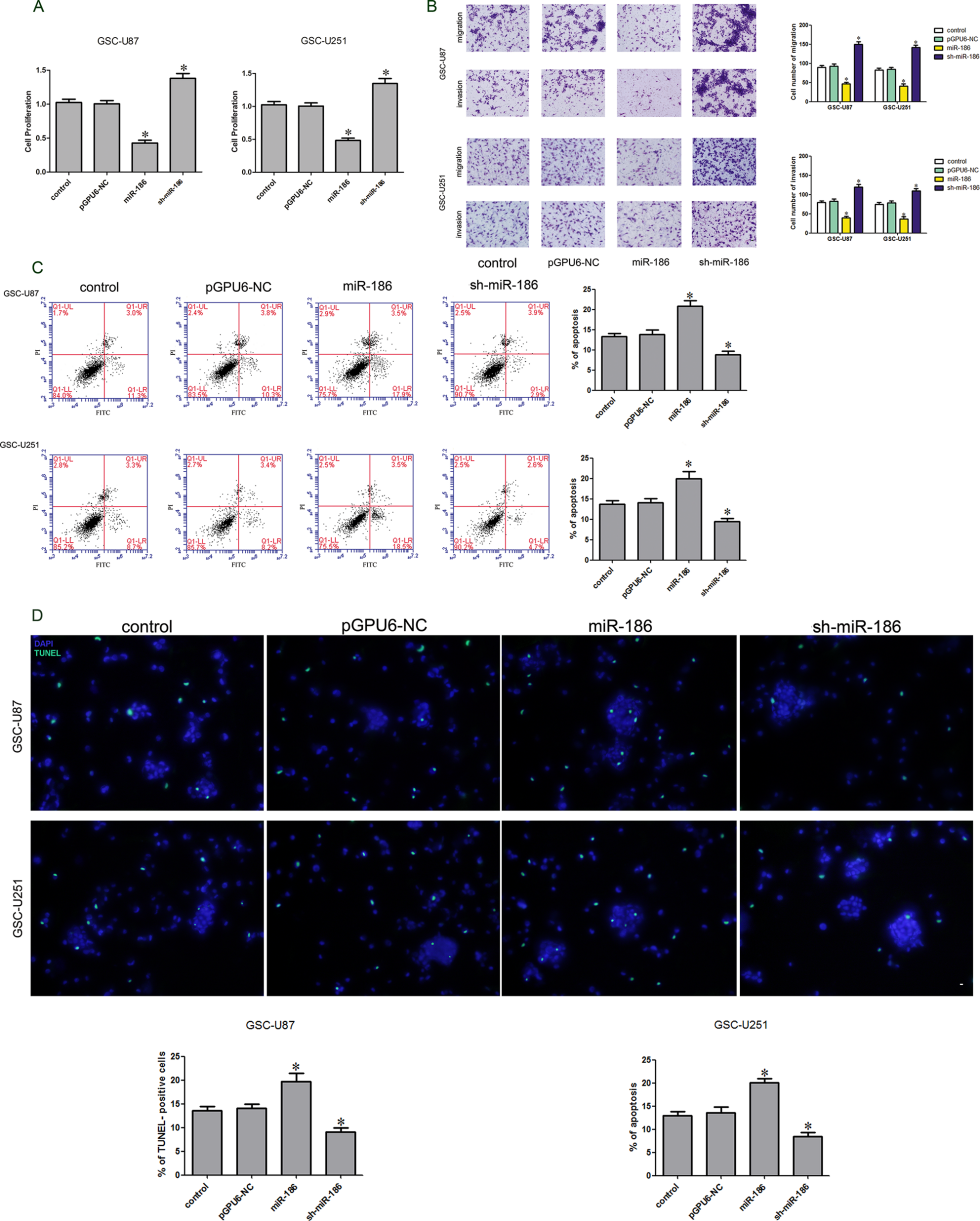
Effect of miR-186 on proliferation, apoptosis, migration and invasion of GSCs **A.** CCK-8 assay was applied to evaluate the proliferation effect of miR-186 on GSCs. **B.** Quantification of migration and invasion cells with the expression of miR-186 changed. Representative images and accompanying statistical plots were presented. **C.** Flow cytometry analysis of GSCs with the expression of miR-186 changed. **D.** Representative images of experiments of TUNEL assay (green) of GSCs with the expression of miR-186 changed. Data are presented as the mean ± SD (*n* = 5, each group). *P < 0.05 vs. pGPU6-NC group. Scale bars represent 20 μm.

As previously described, miR-186 was lowly expressed in glioma tissue and GSCs. The stably transfected stem cell lines with overexpression or knockdown of miR-186 were established. CCK-8 kit was used to detect the cell proliferation. When compared to the pGPU6-NC group, the proliferation rate was significantly increased in the sh-miR-186 group but was significantly decreased in the miR-186 group (Figure 3A). Flow cytometry analysis results showed that the apoptosis rates were 14% ± 1.31%, 20% ± 1.65% and 8% ± 1.19% in pGPU6-NC, miR-186 over-expression and miR-186 knockdown groups respectively (Figure 3C). TUNEL assay was used to further assess the apoptosis. Results showed that the TUNEL-positive cells (%) in the sh-miR-186 group was significantly decreased compared with the pGPU6 group, whereas TUNEL-positive cells (%) in the miR-186 group was significantly increased (Figure 3D). Numbers of migrating and invading cells in the sh-miR-186 group were significantly increased compared with the pGPU6-NC group, whereas migration and invasion were significantly attenuated in the miR-186 group (Figure 3B). In contrary to CRNDE, miR-186 might serve as a tumor suppressor and affect the proliferation, migration, invasion and apoptosis of the GSCs.

### CRNDE bound to and negativelyregulated miR-186

**Figure 4:**
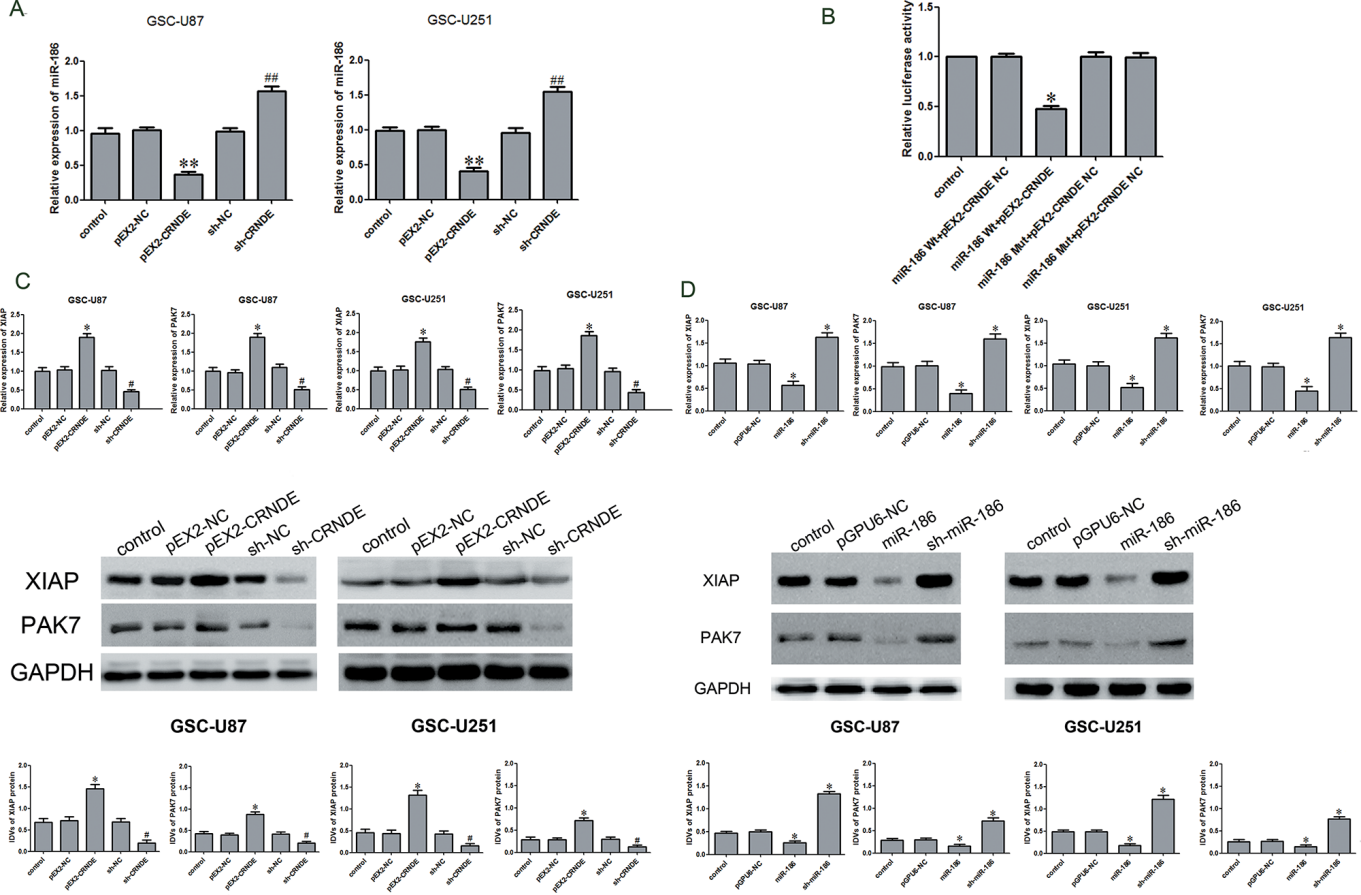
Over-expression of CRNDE inhibited miR-186 expression, down regulation of CRNDE or over-expression of miR-186 inhibited the expression of XIAP and PAK7 **A.** qRT-PCR analysis for CRNDE regulated miR-186 expression in GSCs. Data are presented as the mean ± SD (*n* = 5, each group).**P < 0.01 vs. pEX-NC group; ^##^*P* < 0.01 vs. sh-NC group. **B.** Luciferase reporter assay of HEK 293T cells co-transfected with pEX2-CRNDE or pEX2-CRNDE-NC and miR-186-3′UTR-Wt(miR-186 WT) or the miR-186-3′UTR-Mut(miR-186 Mut). Data are presented as the mean ± SD (*n* = 5, each group). *P < 0.05 vs. XIAP-WT & miR-186 NC group and PAK7-WT & miR-186 NC group. **C.** qRT-PCR and western blot analysis for CRNDE regulated XIAP and PAK7 expression in GSC-U87 and GSC-U251. The relative expression of XIAP and PAK7 are shown using GAPDH as an endogenous control. The IDVs of XIAP and PAK7 are shown using GAPDH as an endogenous control. Data are presented as the mean ± SD (*n* = 5, each group). *P < 0.05 vs. pEX2-NC group; ^#^*P* < 0.05 vs. sh-NC group. **D.** qRT-PCR and western blot analysis for miR-186 regulated XIAP and PAK7 expression in GSC-U87 and GSC-U251. The relative expression of XIAP and PAK7 are shown using GAPDH as an endogenous control. The IDVs of XIAP and PAK7 are shown using GAPDH as an endogenous control. Data are presented as the mean ± SD (*n* = 5, each group). *P < 0.05 vs. pGPU6-NC group.

According to the bioinformatics databases (RNAhybrid), we predicted that CRNDE might be associated with the miR-186 binding sites. Furthermore, dual-luciferase gene reporter assay proved that CRNDE could bind to miR-186 at the predicted binding sites. Quantitative RT-PCR results demonstrated that miR-186 expression was down-regulated in the pEX2-CRNDE group compared with the pEX2-NC group, whereas it was up-regulated in the sh-CRNDE group compared to the sh-CRNDE NC group (Figure 4A). Results of dual-luciferase gene reporter assay showed that the luciferase activity in the pEX-2-CRNDE+miR-186-3′UTR-Wt group was lower than that in the pEX-2-CRNDE-NC+miR-186-NC group (Figure 4B), indicating that CRNDE binded to miR-186 and negatively regulated its expression.

### Overexpression of CRNDE or knockdown of miR-186 regulated the biological behavior of GSCs by regulating XIAP and PAK7

The above results have shown that CRNDE or miR-186 might be involved in the regulation of the biological behavior of GSCs. To further explore the mechanisms, qRT-PCR and Western blot assays were performed to detect the effects of CRNDE or miR-186 on the mRNA and protein expression levels of XIAP and PAK7. The mRNA expression levels of XIAP and PAK7 in the pEX2-CRNDE group were higher than those in the pEX2-NC group (Figure 4C). However the mRNA expression of XIAP and PAK7 in the sh-CRNDE group was down-regulated compared with the sh-NC group. The miR-186 group had a lower expression level of XIAP and PAK7 mRNA than pGPU6-NC group, while the sh-miR-186 group showed the contrary results. The XIAP and PAK7 protein expression levels were determined by Western blot analysis at different expression levels of CRNDE or miR-186 in GSCs (Figure 4B). The results were similar to the findings of qRT-PCR. Compared with the pEX2-NC group, the expression of XIAP and PAK7 protein was up-regulated in the pEX2-CRNDE group, and was down-regulated in the sh-CRNDE group. Compared with the pGPU6-NC group, the expression of XIAP and PAK7 protein was down-regulated in the miR-186 group but was upregulated in the sh-miR-186 group.

### CRNDE attenuated miR-186-induced inhibition of XIAP and PAK7 and affected the biological behavior of GSCs through negative regulation of miR-186

**Figure 5:**
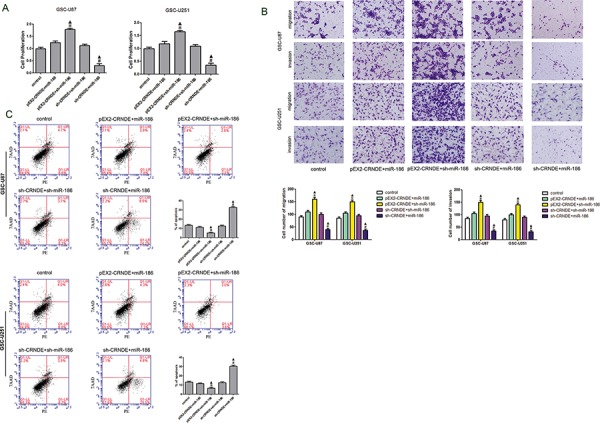

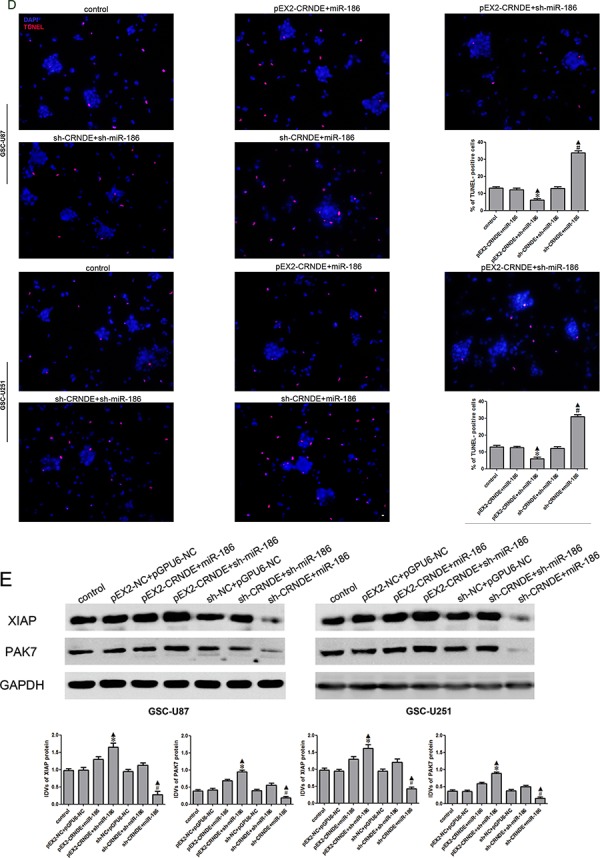
Effect of CRNDE and miR-186 on proliferation, migration, invasion and apoptosis on GSCs and over-expression CRNDE elevated levels of the expression of XIAP and PAK7 by downregulating miR-186 **A.** CCK-8 assay was applied to evaluate the proliferation effect of CRNDE and miR-186 on GSCs. Data are presented as the mean ± SD (*n* = 5, each group). *P < 0.05 vs. pEX2-CRNDE+miR-186 group; ^#^*P* < 0.05 vs. sh-CRNDE+sh-miR-186 group; ^▲^*P* < 0.05 vs. control group. **B.** Quantification of migration and invasion cells with the expression of CRNDE and miR-186 changed. Representative images and accompanying statistical plots were presented. Data are presented as the mean ± SD (*n* = 5, each group). *P < 0.05 vs. pEX2-CRNDE+miR-186 group; ^#^*P* < 0.05 vs. sh-CRNDE+sh-miR-186 group; ^▲^*P* < 0.05 vs. control group. Scale bars represent 20 μm. **C.** Flow cytometry analysis of GSC-U87 and GSC-U251 with the expression of CRNDE and miR-186 changed. **D.** Representative images of experiments of TUNEL assay (red) of GSCs with the expression of with the expression of CRNDE and miR-186 changed. Data are presented as the mean ± SD (*n* = 5, each group). *P < 0.05 vs. pEX2-CRNDE+miR-186 group; ^#^*P* < 0.05 vs. sh-CRNDE+sh-miR-186 group; ^▲^*P* < 0.05 vs. control group. Scale bars represent 20 μm. **E.** Western blot analysis for CRNDE and miR-186 regulated IDVs of XIAP and PAK7, they are shown using GAPDH as endogenous control. Data are presented as the mean ± SD (*n* = 5, each group). *P < 0.05 vs. pEX2-CRNDE+miR-186 group; ^#^*P* < 0.05 vs. sh-CRNDE+sh-miR-186 group; ^▲^*P* < 0.05 vs. control group.

To verify whether CRNDE was involved in the regulation of XIAP and PAK7 expression as well as the biological behavior of GSCs through the miR-186 pathway, GSCs were transfected with over-express or down-regulate CRNDE and miR-186. The proliferation rate in the pEX2-CRNDE+sh-miR-186 group was increased compared with the pEX2-CRNDE+miR-186 group, whereas it was decreased in the sh-CRNDE+miR-186 group compared with the sh-CRNDE+sh-miR-186 group (Figure 5A). This result implied that CRNDE regulated GSC proliferation through miR-186 pathway. The apoptotic rate was significantly increased in the sh-CRNDE+sh-miR-186 and sh-CRNDE+miR-186 groups (Figure 5C). Furthermore, the pEX2-CRNDE+sh-miR-186 group had a significant lower apoptotic rate than pEX2-CRNDE+miR-186 group, suggesting that CRNDE inhibited miR-186 expression and further prevented the apoptosis of GSCs. TUNEL assay was used to further assess the apoptosis. Results showed that the TUNEL-positive cells (%) in the pEX2-CRNDE+sh-miR-186 group was significantly decreased compared with the pEX2-NC group, whereas TUNEL-positive cells (%) in the sh-CRNDE+miR-186 group was significantly increased (Figure 5D). In addition, the effect of CRNDE on the migration and invasion of GSCs was also explored (Figure 5B). Results showed that numbers of migrating and invading cells in the pEX2-CRNDE+sh-miR-186 group were significantly increased compared with the pEX2-CRNDE+miR-186 group. However the numbers of migrating and invading GSCs in the sh-CRNDE+miR-186 group were significantly lower than those in the sh-CRNDE+sh-miR-186 group. Western blot analysis was performed to detect the protein expression levels of XIAP and PAK7 in the GSCs at different expression levels of CRNDE and miR-186. XIAP and PAK7 protein expression levels in the pEX2-CRNDE+sh-miR-186 group were significantly increased compared with the pEX2-CRNDE+miR-186 group, while they were significantly decreased in the sh-CRNDE+miR-186 group compared with the sh-CRNDE+sh-miR-186 group (Figure 5D), indicating that CRNDE inhibited miR-186 expression and accordingly regulated the biological behavior of GSCs.

### miR-186 bound to the 3′UTR of XIAP and PAK7 and regulated the expression of caspase 3, BAD, cyclin D1 and MARK2

**Figure 6:**
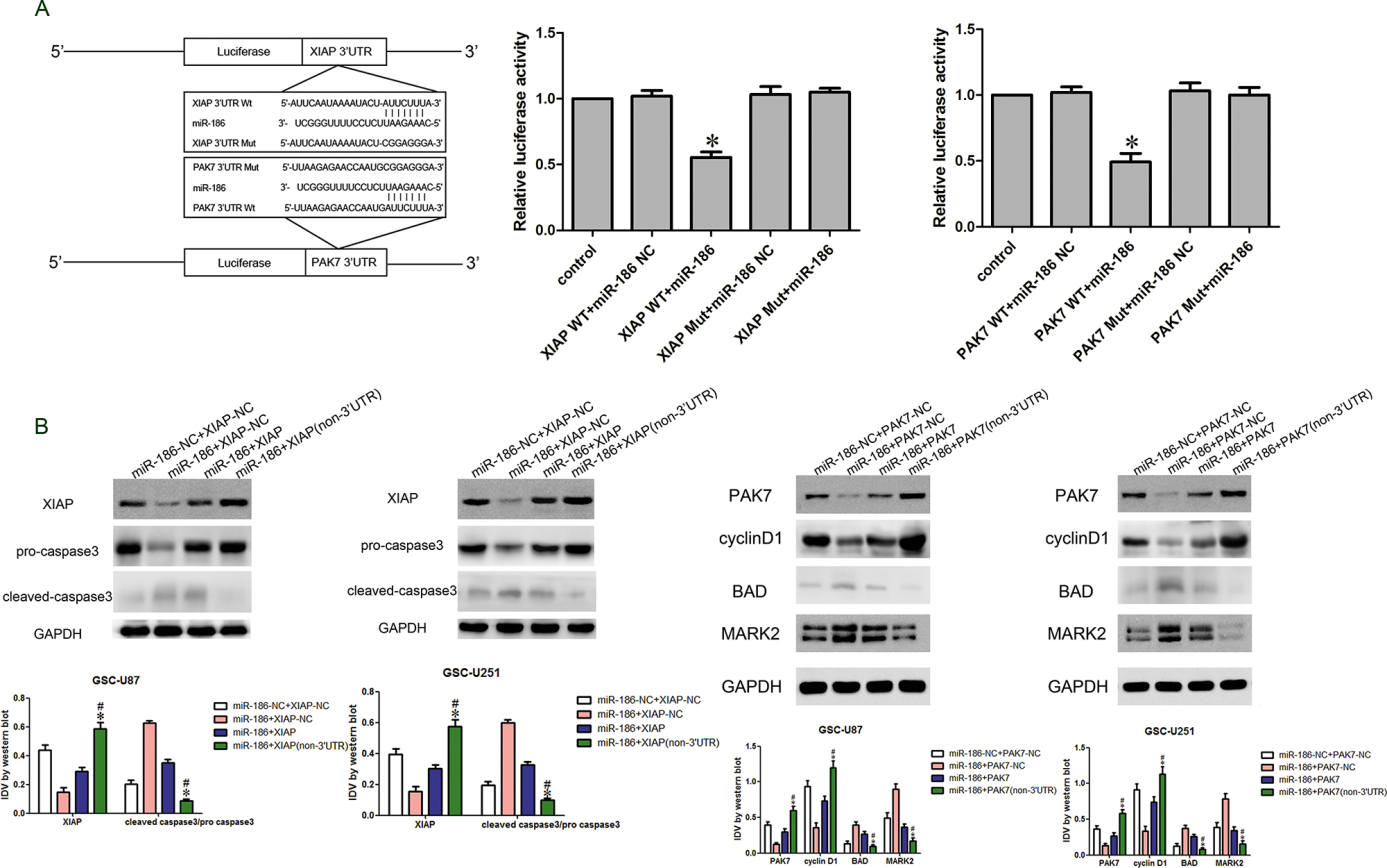
miR-186 regulated caspase3, cyclin D1, BAD and MARK2 by targeting XIAP and PAK7′s 3′-UTR **A.** The predicted miR-186 binding sites in the 3′-UTR region of XIAP (XIAP-3′-UTR-Wt) or PAK7 (PAK7-3′-UTR-Wt) and the designed mutant sequence (XIAP-3′UTR-Mut or PAK7-3′UTR-Mut) were indicated. Luciferase reporter assay of HEK 293T cells transfected with XIAP-3′UTR-Wt (XIAP WT) (or the XIAP-3′UTR-Mut (XIAP Mut))/PAK7-3′UTR-Wt (PAK7 WT) (or the PAK7-3′UTR-Mut (PAK7 Mut)) and the indicated miRNAs. Data are presented as the mean ± SD (*n* = 5, each group). *P < 0.05 vs. XIAP-WT & miR-186 NC group and PAK7-WT & miR-186 NC group. **B.** Western blot analysis of the pro-caspase3 and cleaved caspase3 regulated by miR-186 and XIAP in GSC-U87 and GSC-U251. Data are presented as the mean ± SD (*n* = 5, each group). *P < 0.05 vs. miR-186+XIAP group; ^#^*P* < 0.05 vs. miR-186+XIAP-NC group. **C.** Western blot analysis of the cyclin D1, BAD and MARK2 regulated by miR-186 and PAK7 in GSC-U87 and GSC-U251. Data are presented as the mean ± SD (*n* = 5, each group). *P < 0.05 vs. miR-186+PAK7 group; ^#^*P* < 0.05 vs. miR-186+PAK7-NC group.

According to the bioinformatics database (Targetscan, Pictar, miRanda), miR-186 might bind to XIAP and PAK7 3′UTR region. Dual-luciferase reporter assay results showed that the luciferase activity in the miR-186+XIAP Wt or miR-186+PAK7 Wt group was significantly lower than that in miR-186-NC+XIAP Wt or miR-186-NC+PAK7 Wt group, while miR-186 did not affect the luciferase activity in XIAP Mut or PAK7 Mut group (Figure 6A). Western blot analysis was performed to determine whether miR-186 could regulate the expression of XIAP, PAK7, caspase3, BAD, cyclin D1 and MARK2. The protein expression levels of pro-caspase3 in the miR-186+XIAP (non-3′UTR) group were significantly increased, while the expression of cleaved caspase 3 was significantly decreased compared with the miR-186+XIAP group (Figure 6B). Similarly, the protein expression levels of MARK2 and BAD in the miR-186+PAK7 group were decreased, while cyclin D1 was significantly up-regulated compared with the miR-186+PAK7 group (Figure 6C).

### Knockdown of CRNDE combined with overexpression of miR-186 significantly inhibited tumor growth *in vivo*

**Figure 7:**
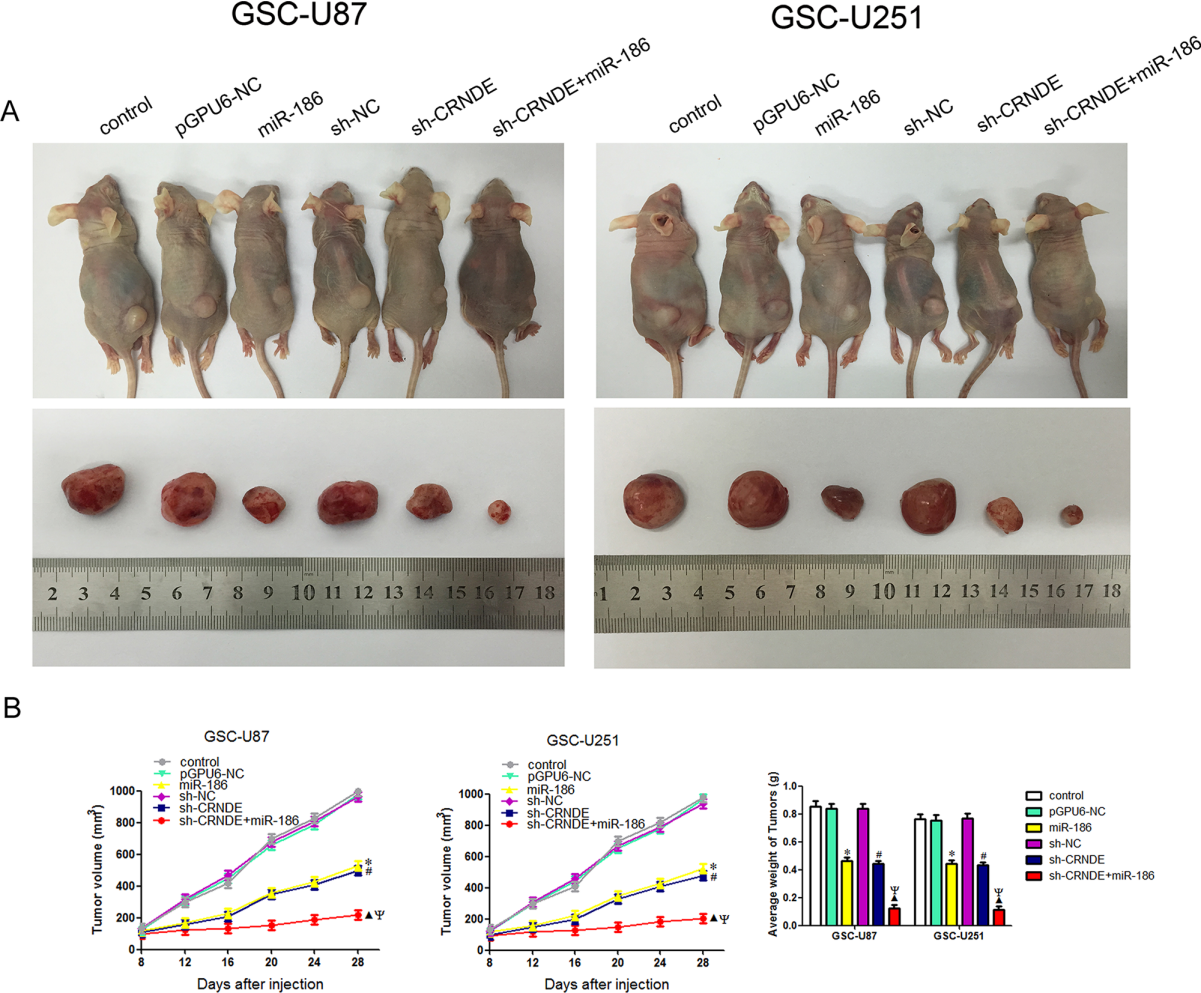
*In vivo* tumor xenografts study **A.** The stable expressing cells were used for the *in vivo* study. The nude mice carrying tumors from respective groups were shown. The sample tumor from respective group was shown. **B.** Tumor volume was calculated every four days after injection, and the tumor was excised and weighed after 28 days. *P < 0.05 vs. pGPU6-NC group; ^#^*P* < 0.05 vs. sh-NC group; ^▲^*P* < 0.05 vs. miR-186 group; ^Ψ^*P* < 0.05 vs. sh-CRNDE group.

To determine the functions of CRNDE and miR-186 *in vivo*, we analyzed the effects of CRNDE knockdown, miR-186 overexpression and their combination on the glioma growth in tumor-bearing nude mice. From 2^th^ to 4^th^ week, sh-CRNDE group and miR-186 group developed significantly smaller tumors than pEX2-NC group and pGPU6-NC group. And in co-transfected sh-CRNDE+miR-186 group, the volume of the tumor was smaller compared with either sh-CRNDE or miR-186 group (Figure 7A). The volumes and weights of transplanted tumors were reduced in the sh-CRNDE group or miR-186 group compared with control group, and in co-transfected sh-CRNDE+miR-186 group, the inhibition of transplanted tumors were enhanced compared with either sh-CRNDE or miR-186 group alone (Figure 7A, 7B).

## DISCUSSION

GSCs are a subgroup of glioma cells that are characterized as self-renewing, angiogenesis-promoting and multi-differentiational cells. The effects of conventional therapies against glioma may be limited due to the easy recurrence, drug-resistance, rapid growth, invasion and metastasis [16]. The refractoriness of glioma is partly caused by the existence of GSCs. Therefore, therapeutics targeting GSCs has become a hot topic in field of glioma treatment. The present study showed that CRNDE was highly expressed in the GSCs, while miR-186 was lowly expressed in glioma tissue and GSCs. Moreover, we found that CRNDE bound to miR-186 and negatively regulated its expression. Furthermore, CRNDE regulated the proliferation, migration, invasion and apoptosis of GSCs. Moreover, MiR-186 could also regulate the expression of caspase3, BAD, cyclin D1 and MARK2 by binding to the target genes and negatively regulating XIAP and PAK7.

Increasing evidence has shown that lncRNAs are abnormally expressed in tumors and play a crucial role in the biological processes of tumor cells. Mazar J. *et al*. found that lncRNA-SPRY4-IT1 promoted the invasion and proliferation of human melanoma cells and inhibited the apoptosis through MARK pathway [17]. Lloyd D. Graham *et al*. demonstrated that CRNDE served as a cancer-promoting gene in colorectal carcinoma and promoted the growth of colorectal carcinoma cells [5]. CRNDE also contributed to the proliferation, migration and invasion of colorectal cancer cell. Blake C. Ellis *et al*. found that the expression level of CRNDE was higher in glioma tissue than that in normal brain tissue [7]. However, the functions of CRNDE in glioma still remain unclear. To further explore the role of CRNDE in GSCs, we performed quantitative RT-PCR and found that CRNDE was highly expressed in GSCs compared with the non-stem cells, suggesting that CRNDE might serve as an oncogene in GSCs. In addition, results of the present study showed that CRNDE not only promoted cell proliferation, invasion, migration but also inhibited apoptosis in GSCs, which was consistent with the results of colorectal cancer cells.

MiRNAs have various expression patterns and biological functions in different tumors. MiR-219 regulates PRKCI and accordingly inhibits the proliferation of human tongue squamous cell carcinoma [18]. MiR-101 regulates zeste homolog 2 and further inhibits the growth of bladder cancer stem cells [19]. In gliomas, miRNAs might act as either tumor promoter or suppressor. MiR-18a promotes the proliferation, migration and invasion of glioma cells, whereas miR-146a and miR-449a inhibit glioma growth by inducing cellular apoptosis [20–22]. However, the expression and functions of miR-186 in glioma are poorly understood. The present study showed that miR-186 was lowly expressed in glioma tissue compared to normal brain tissue, and the expression was also lower in the GSCs than that in the non-stem cells. In addition, miR-186 was found to inhibit the proliferation, migration, invasion and promoted apoptosis in GSCs. This evidence indicated that miR-186 could be regarded as a tumor suppressor gene that inhibited the biological behavior of GSCs, which was consistent with the results observed in non-small cell lung cancer.

The aforementioned experiments demonstrated the controversial functions of CRNDE and miR-186 in GSCs. During the investigation of the relationship between the two factors in regulating GSCs, we found a binding site between CRNDE and miR-186 according to bioinformatics database (RNAhybrid). As shown in figure 4A, the expression level of endogenous miR-186 was negatively correlated with the CRNDE. Results of dual-luciferase reporter assay showed that CRNDE was capable of binding to miR-186, suggesting that CRNDE might affect the biology of GSCs by regulating miR-186.

XIAP is the strongest apoptosis-inhibitor in the IAPs family, which is located at Xq25 and is critically involved in the tumor-tolerant radiotherapy and chemotherapy [23, 24]. Over the past decades, growing evidence has shown that XIAP functions to promote the tumor growth and to inhibit the apoptosis in a variety of tumor cells [25–27]. U Naumann *et al*. found that XIAP was up-regulated in glioma tissues and itself regulated the expression of BCL-2 gene family by activating NF-κB pathway, resulting in promoted proliferation and inhibited apoptosis in U87MG cells [13]. P21-activated kinase 7 (PAK7, also known as PAK5) is a recently discovered member in the P21-activated protein kinase family. PAK7 is mainly expressed in brain and located in the mitochondria, whose N and C-terminals are CDC42/Rac1 and Ste20-like [28]. PAK7 plays an important role in various cellular functions, such as cytoskeletal reorganization, cell growth, proliferation, differentiation, apoptosis and gene transcription [29]. Recent studies have confirmed that PAK7 was highly expressed in tumor cells (particularly in glioma cells), and was involved in tumor formation, metastasis, migration and infiltration through multiple signaling pathways [30–32]. In summary, XIAP and PAK7 are involved in the important biological processes of glioma cells. To verify whether the two factors were involved in the CRNDE or miR-186-induced regulation of GSC biological behavior, we transfected GSCs to alter the expression of CRNDE and miR-186. Results showed that the overexpression of CRNDE increased the mRNA and protein expression levels of XIAP and PAK7, whereas the overexpression of miR-186 reduced their expression levels. Experimental findings indicated that CRNDE and miR-186 can exert biological effects by regulating XIAP and PAK7 expression.

LncRNAs can regulate the miRNAs expression and most of their functions are mediated by the negative regulation of miRNAs. Zhang H *et al*. found that HOTAIR negatively regulated miR-7 expression and promoted STAT3 and SETDB1 expression, thus promoting the proliferation and invasion of breast cancer stem cells [33]. As previously described, CRNED bound to and negatively regulated miR-186 and both factors affected the biological characteristics of GSCs through regulating the expression of XIAP and PAK7. We then investigated whether CRNDE-induced negative regulation of miR-186 would influence the biological characteristics of GSCs and found that the overexpression of CRNDE and knockdown of miR-186 promoted the proliferation, migration and invasion while inhibited the apoptosis in GCSs. The combination of CRNDE overexpression and miR-186 knockdown produced higher XIAP and PAK7 expression levels than those in either CRNDE or miR-186 overexpression group alone. Results of *in vivo* studies showed that the overexpression of CRNDE combined with knockdown of miR-186 reduced the tumor volumes and weights of tumor-bearing nude mice. In summary, CRNDE could bind to miR-186 and negatively regulate its expression, which contributes to the regulation of GSC biological properties. This is also the underlying mechanism by which CRNDE regulates the biology of GSCs.

**Figure 8:**
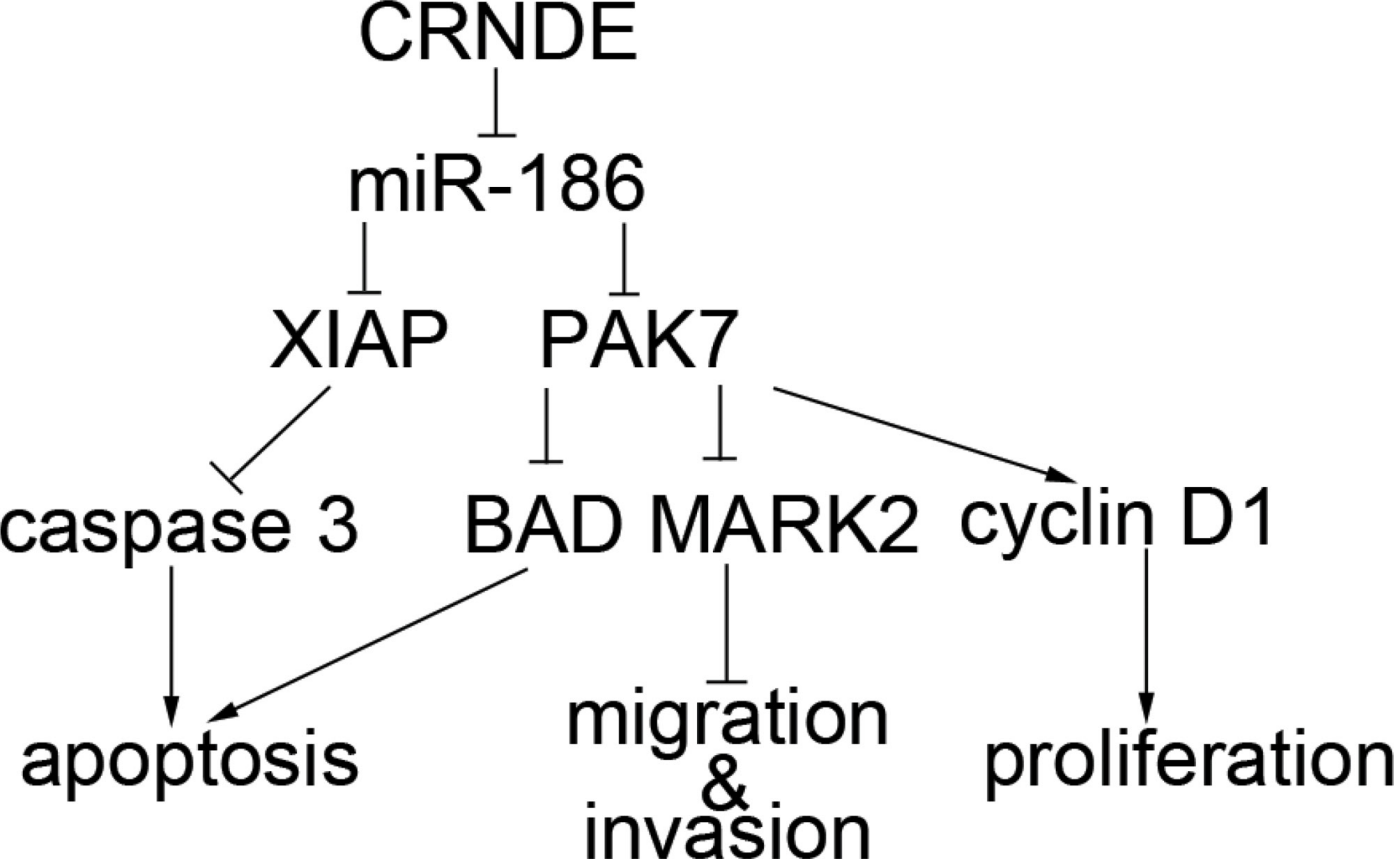
The schematic cartoon of the mechanism of CRNDE as a oncogene negative regulation of miR-186 of GSCs

MiRNAs act as regulatory factors through targeting the 3′UTR region of taget mRNAs in cells [34, 35]. MiR-186 3′UTR region was identified as the binding site between PAK7 and XIAP by the biological softwares (Targetscan, Pictar, miRanda). Our hypothesis was confirmed by the dual-luciferase reporter assay, suggesting that miR-186 possibly affected the biological behavior of GSCs through the negative regulation of XIAP and PAK7. Furthermore, we found that miR-186 might negatively regulate XIAP and PAK7, thus affecting the expression levels of caspase3, BAD, cyclin D1 and MARK2. Caspase 3 is a direct and the strongest pro-apoptotic enzyme in the cysteine protease family. XIAP has three BIR structures, BIR1, BIR2 (binds to caspase 3) and BIR3. The RING structural domain of XIAP has E3 ubiquitin ligase activity, which contributes to protein ubiquitination and accelerates protein degradation. It may promote caspase3 degradation and thus weakens its pro-apoptotic effect. Meanwhile, XIAP binds to the substrate of capase3 and inhibits the pro-apoptosis effect [24, 36, 37]. BAD is a pro-apoptotic gene associated with Bcl-2 and Bcl-xL in the Bcl-2 family. It is highly involved in a variety of apoptotic pathways and functions as a pro-apoptotic factor through the positioning of mitochondria. PAK7 inhibits the apoptosis through detaching BAD from the mitochondria and reducing its total quantity [38, 39]. Cyclin D1 belongs to the cyclin protein family and has three subtypes: D1, D2, and D3. Among them, D1 is strongly associated with cancer. Cyclin D1 is dominant at G0/G1 phase and is synthesized at G1 phase. Then it forms cyclin D1-CDK4 or cyclin D1-CDK6 complexes, activates CDK, and phosphorylates the key substrate retinoblastoma gene (Rb), which enables the cells to enter S phase through the restriction point. The up-regulated expression of cyclin D1 promotes the phosphorylation of pRb and accelerates the entry into S phase, resulting in promoted cell proliferation [40]. PAK7 up-regulates the cyclin D1 expression and promotes tumor cell proliferation [41]. MARK2 is a class of kinases that regulate cell polarity. It can detach the tau protein from the cell microtubules where tau protein is tightly conjugated and phosphorylate it. PAK7 binds to the catalytic domain of MARK2 without phosphorylating it. PAK7 regulates cell adhesion and the morphology of stress fiber by down-regulating MARK2 expression, leading to changes of GSCs migration and invasion [42–44]. Results of the present study showed that, the pro-caspase 3 protein expression was significantly increased while cleaved caspase3 protein expression was significantly decreased in the miR-186+XIAP (non-3′UTR) transfected GSCs compared to the miR-186+XIAP transfected GSCs. We further investigated whether miR-186 affected the GSC properties by regulating PAK7 and found that the miR-186+PAK7(non-3′UTR)-transfected GSCs had significantly higher expression levels of BAD and MARK2 proteins and lower expression level of cyclin D1 protein than the miR-186+PAK7-transfected GSCs. This evidence indicated that miR-186 played a role in regulating the expression levels of caspase 3, BAD, cyclin D1 and MARK2 through negative regulation of XIAP and PAK7 expression, which might be the mechanism in the regulation of the biological behavior of GSCs. The mechanism underlying the suppression of GSCs by down-regulating of CRNDE is schematically presented in Figure 8.

In summary, this is the first study that discovered and proved that CRNDE was up-regulated while miR-186 was down-regulated in GSCs. CRNDE could promote GSCs proliferation, migration, invasion and inhibit GSCs apoptosis by negatively regulating miR-186. MiR-186 could bind to XIAP and PAK7 3′UTR and accordingly played a negative role on regulating the expression of caspase3, BAD, cyclin D1 and MARK2. Therefore, we suggested that CRNDE/miR-186 played an important role in GSCs and the present study provided evidence for novel targets in glioma treatment.

## MATERIALS AND METHODS

### Human tissue samples and cell culture

Glioma tissues and normal brain tissues (NBTs) were collected form patients undergoing surgery at the Department of Neurosurgery, Shengjing Hospital of China Medical of University. Parts of the fresh glioma tissues were sent for routine neuropathological evaluation after surgery resection, the rest were put in liquid nitrogen or used for the ensuing experiments. Informed consents were obtained from all patients and the study was approved by the Ethics Committee of Shengjing Hospital of China Medical University. Grades of glioma were evaluated according to WHO classification by neuropathologists. Human GBM cell lines (U87 and U251) were obtained from the Department of Neurobiology, College of Basic Medicine, China Medical University. Human embryonic kidney (HEK) 293T cells were obtained from Shanghai Institutes for Biological Sciences Cell Resource Center. They were cultured in Dulbecco's Modified Eagle Medium (DMEM) of high glucose with 10% fetal bovine serum (FBS, Gibco, Carlsbad, CA, USA). All cells were incubated at 37°C in a humidified incubator with 5% CO_2_.

### Isolation and identification of GSCs

Glioma stem cells (GSCs) were obtained and isolated as described previously [22, 45, 46]. GSCs were resuspended in DMEM/F-12 medium (Life Technologies Corporation, Grand Island, NY, USA) supplemented with basic fibroblast growth factor (bFGF, 20ng/ml, Life Technologies Corporation, Carlsbad, CA, USA), epidermal growth factor (EGF, 20 ng/ml, Life Technologies Corporation, Gaithersburg, MD, USA) and 2% B27 (Life Technologies Corporation, Grand Island, NY, USA).

### Reverse transcription and quantitative real-time PCR (qRT-PCR)

Total RNA were extracted from cells using Trizol reagent (Life Technologies Corporation, Carlsbad, CA, USA). RNA concentration and quality were determined by the 260/280 nm ratio using a Nanodrop Spectrophotometer (ND-100). One-Step SYBR PrimeScript RT-PCR Kit (Perfect Real Time) (Takara Bio, Inc, Japan) was used for qRT-PCR. The primers for CRNDE: 5′-ATATTCAGCCGTTGGTCTTTGA-3′, 5′-TCTGCGTGACAACTGAGGATTT-3′; XIAP:5′-CCTC AAAGTCAAAGCAAATCG-3′, 5′-AGGTAGGAAGCCG TGGAGAT-3′; for PAK7: 5′-AGACCATCCTGGCTA ACACG-3′, 5′-GGGTTCACACCATTCTCCTG-3′ and for glyceraldehyde-3-phosphate dehydrogenase (GAPDH): 5′-GGTGAAGGTCGGAGTCAACG-3′, 5′-CCATGTAGT TGAGGTCAATGA AG-3′. TaqMan MicroRNA Reverse Transcription kit (Applied Biosystems, Foster City, CA, USA) was used for miRNA reverse transcription and qRT-PCR was conducted using TaqMan Universal Master Mix II with TaqMan microRNA assays of miR-186-5p and U6 respectively. U6 and GAPDH were used as endogenous controls for miRNA and gene expression detection. Expression were normalized to endogenous controls and relative quantification (2^−ΔΔ^Ct) method was used for fold changes' calculating.

### Cell transfections

CRNDE full length (pEX2-CRNDE) plasmid, four hairpin CRNDE (sh-CRNDE, CRNDE-homo-420, CRNDE-homo-452, CRNDE-homo-572, CRNDE-homo-787) plasmids and their respective non-targeting sequence (negative control, NC); miR-186-5p plasmid, sh-miR-186-5p plasmid and their respective non-targeting sequence (negative control, NC) were synthesized (GenePharma, Shanghai, China). XIAP full length(with 3′UTR) plasmid, XIAP(without 3′UTR) plasmid and their respective non-targeting sequence (negative control, NC);PAK7 full length(with 3′UTR) plasmid, PAK7 (without 3′UTR) plasmid and their respective non-targeting sequence (negative control, NC) were synthesized (Life technology, MA, USA). The sequence of sh-CRNDE was 5′-CACCGGAAGGAGGAGATTCTGAAGATTCAAGA GATCTTCAGAATCTCCTCCTTCCTTTTTG-3′, 5′-GAT CCAAAAAAGGAAGGAGGAGATTCTGAAGATCTCT TGAATCTTCAGAATCTCCTCCTTCC-3′. The sequence of sh-NC was:

5′-CACCGTTCTCCGAACGTGTCACGTCAAGA GATTACGTGACACGTTCGGAGAATTTTTTG-3′, 5′-G ATCCAAAAAATTCTCCGAACGTGTCACGTAATCTC TTGACGTGACACGTTCGGAGAAC-3′. The sequence of miR-186-5p was 5′-CACCGCAAAGAATTCTCC TTTTG GGCTTTCAAGAGAAGCCCAAAGAGAATTC TTTG TTTTTG-3′, 5′-GATCCAAAAAACAAAGAATT CTCTTTGGGCTTC TCTTGAAAGCCCAAAAGGAGA ATTCTTTGC-3′. The sequence of sh-miR-186-5p was: 5′-CACCGCGCCCAA AAGGAGAATTCTTTGTT CAAGAGACAAAGAATTCCTTTTGGGCTTTTTTT G-3′, 5′-GATCCAAAAAAAGC CCAAAAGGAATTCT TTGTCTCTTGAACAAAGAATTCTCCTTTTGGGCT C-3′. The sequence of the negative control of miR-186-5p and sh-miR-186-5p (pGPU6-NC) was: 5′-CACCGTTCTCCGAACGTGTGT CACGTCACGT CAAGAGATTACGTGACACGTTCGGAGAATTTTTG -3′, 5′-GATCCAAAAAATTCTCCGAA CGTGTCACG TGTCACGTAATCTCTTGACGTGACACGTTCGGAG AA C-3′. Cells were seeded into 24-well plates (Corning) until they were at 50–70% confluence and then transfected using Opti-MEM I and Lipofectamine 2000 reagent (Life Technologies Corporation, Carlsbad, CA, USA). After 6 h of transfection, the medium was replaced with high-glucose DMEM medium with 10% FBS. The applicable stably transfected cells were selected using G418 screening. The overexpression and the silence efficiency were analyzed using qRT-PCR. And then GSCs were isolated as previously described. To determine the effect of CRNDE on GSCs, cells were divided into five groups: control group, pEX2-NC group (tansfected with empty plasmid), pEX2-CRNDE group, sh-NC group and sh-CRNDE group. To determine the effect of miR-186-5p on GSCs, cells were divided into four groups: control group, pGPU6-NC (also shown as miR-186 NC) (transfected with empty plasmid), miR-186 group (transfected with miR-186-5p plasmid), sh-miR-186 group (transfected with sh-miR-186-5p plasmid). To determine whether CRNDE-mediated regulation of miR-186 expression could regulate the behavior of GSCs, cells were divided into seven groups: control group, pEX2-NC+pGPU6-NC group (transfected with both pEX2-NC and pGPU6-NC), pEX2-CRNDE+miR-186 group (transfected with both pEX2-CRNDE and miR-186-5p), pEX2-CRNDE+sh-miR-186 group (transfected with both pEX2-CRNDE and sh-miR-186-5p), sh-NC+pGPU6-NC group (transfected with both sh-NC and pGPU6-NC), sh-CRNDE+sh-miR-186 group (transfected with both sh-CRNDE and sh-miR-186-5p), sh-CRNDE+miR-186 group (transfected with both sh-CRNDE and miR-186-5p). To determine whether miR-186 could target XIAP and PAK7 on their 3′UTR region, and affect GSCs' behavior by regulating the expression of caspase3, cyclin D1, BAD and MARK2, cells were divided into four groups:miR-186-NC+XIAP-NC group (transfected with both pGPU6 and empty plasmid of XIAP), miR-186+XIAP-NC group (transfected with both miR-186-5p and empty plasmid of XIAP), miR-186+XIAP group (transfected with both miR-186-5p and XIAP full length plasmid (with 3′UTR)), miR-186+XIAP (non-3′UTR) group (transfected with both miR-186-5p and XIAP without 3′UTR plasmid); miR-186-NC+PAK7-NC group (transfected with both pGPU6 and empty plasmid of PAK7), miR-186+PAK7-NC group (transfected with both miR-186-5p and empty plasmid of PAK7), miR-186+ PAK7 group (transfected with both miR-186-5p and PAK7 full length plasmid (with 3′UTR)), miR-186+PAK7 (non-3′UTR) group (transfected with both miR-186-5p and PAK7 without 3′UTR plasmid).

### Cell proliferation assay

Cell proliferation assays were performed using the Cell Counting Kit-8 (CCK-8, Beyotime Institute of Biotechnology, Jiangsu, China). After transfection, cells were seeded in 96-well plates at the density of 2000/well. After 48 h, 20 μL of CCK-8 were added into per well and incubated at 37°C for 2 h. The absorbance was measured at 450 nm.

### Cell migration and invasion assays

24-well chambers with 8 μm pore size (Corning) were used in cell migration and invasion assays. Cells were resuspended in 100 μL serum-free medium and seeded into the upper chamber (without or pre-coated with 500 ng/ml Matrigel solution (BD, Franklin Lakes, NJ, USA) in migration or invasion assay separately), 600 μL of 10% FBS medium was placed in the lower chamber. After 48 h of incubation, the upper chambers were removed from the plates and cells on the top side of the chamber were wiped with a cotton swab. Migrating or invading cells were fixed and then stained by Giemsa staining. Five randomly fields were counted under a microscope and photos were taken.

### Quantization of apoptosis by flow cytometry

Cell apoptosis was quantified by Annexin V-FITC/PI or Annexin V-PE/7AAD staining (Southern Biotech, Birmingham, AL, USA). After washing with PBS twice, cells were stained with Annexin V-FITC/PI or Annexin V-PE/7AAD according to the manufacturer's instructions. Then cells were analyzed by flow cytometry (FACScan, BD Biosciences) and apoptotic fractions were acquired.

### TUNEL assay

TUNEL assay was performed according to the manufacturer's instructions (Beyotime Institute of Biotechnology). Nuclei were stained with DAPI (Beyotime Institute of Biotechnology). Fluorescence images were visualized using a fluorescent microscope (Olympus, Tokyo, Japan).

Quantification of TUNEL was measured using the percentage of TUNEL-positive cells (red or green cells) relative to total cells (DAPI). Cells were counted from randomly selected five fields.

### Western blot analysis

Total proteins were extracted form the cells using RIPA buffer with protease inhibitors (Beyotime Institute of Biotechnology) on ice, subjected to SDS-PAGE and electrophoretically transferred to PVDF membranes. Membranes were incubated in 5% nonfat milk dissolved in Tris-buffered saline (TBS) containing 0.1% Tween-20 for 3 h at room temperature and then incubated with primary antibodies as follows: XIAP (1:1000, Proteintech, Chicago, IL), PAK7 (1:500, Abcam, EUGENE, USA), pro-caspase3 and cleaved-caspase3 (1:1000, SAB, College Park, Maryland, USA), cyclin D1 (1:500, Santa Cruz Biotechnology), BAD (1:2000, Abcam, EUGENE, USA), MARK2 (1:2000, Novus, Littleton, CO, USA), and GAPDH (1:1000, Santa Cruz Biotechnology), followed by incubation with appropriate correlated HRP-conjugated secondary antibody. Then the membranes were incubated with secondary antibodies (Santa Cruz Biotechnology) at room temperature for 2 h. Immunoblots were visualized by enhanced chemiluminescence (ECL kit, Santa Cruz Biotechnology) and scanned using ChemImager 5500 V2.03 software. The relative integrated density values (IDVs) were calculated based on GAPDH as an internal control.

### Reporter vectors construction and luciferase assays

MiR-186-3′UTR, XIAP 3′-UTR and PAK7 3′-UTR sequences were amplified by PCR and cloned into a pmirGlo Dualluciferase miRNA Target Expression Vector (Promega, Madison, WI, USA) to construct 3′-UTR-luciferase reporter vector (miR-186-WT, XIAP-WT, PAK7-WT) (GenePharma). The sequence of putative binding site was replaced as indicated (miR-186-Mut, XIAP-Mut, PAK7-Mut) to mutate the putative binding site of CRNDE or miR-186 in the 3′-UTR-containing vector. HEK-293T cells were seeded in 96-well plates and the cells were co-transfected with miR-186-WT(or miR-186-Mut) or XIAP-WT (or XIAP-Mut) or PAK7-WT (or PAK7-Mut) and pEX2-CRNDE or miR-186 plasmids when they reached 50–70% confluence. The luciferase activities were measured at 48 h after transfection by Dual-Luciferase reporter assay kit (Promega). The cells were divided in five groups: control group, miR-186 WT+pEX2-CRNDE NC (transfected with miR-186-WT and pEX2-CRNDE-NC), miR-186 WT+ pEX2-CRNDE group (transfected with miR-186-WT and pEX2-CRNDE), miR-186 Mut+ pEX2-CRNDE NC group (transfected with miR-186-Mut and pEX2-CRNDE-NC), miR-186 Mut+ pEX2-CRNDE group (transfected with miR-186-Mut and pEX2-CRNDE); control group, XIAP WT+miR-186 NC (transfected with XIAP-WT and pGPU6-NC), XIAP WT+miR-186 group (transfected with XIAP-WT and miR-186-5p), XIAP Mut+miR-186 NC group (transfected with XIAP-Mut and pGPU6-NC), XIAP Mut+miR-186 group (transfected with XIAP-Mut and miR-186-5p); control group, PAK7 WT+miR-186 NC (transfected with PAK7-WT and pGPU6-NC), PAK7 WT+miR-186 group (transfected with PAK7-WT and miR-186-5p), PAK7 Mut+miR-186 NC group(transfected with PAK7-Mut and pGPU6-NC), PAK7 Mut+miR-186 group(transfected with PAK7-Mut and miR-186-5p)

### Tumor xenograft implantation in nude mice

The stably transfected GSCs were used in the *in vivo* study. Lentivirus encoding miR-186-5p was generated using pLenti6.3/V5eDEST Gateway Vector Kit (Life Technologies Corporation, Carlsbad, CA, USA). The miR-186-5p and short-hairpin RNA targeting human CRNDE were ligated into the pLenti6.3/V5eDEST vector and LV3-CMV-GFP-Puro vector (GenePharma, Shanghai, China), respectively. And then pLenti6.3/V5eDEST-miR-186 and LV3-CMV-GFPPuro-sh-CRNDE vectors were generated. The ViraPower Packaging Mix was used to generate Lentivirus in 293FT cells. After infection, the stable expressing cells of miR-186 and sh-CRNDE were picked. The lentiviruses of miR-186 were transduced in sh-CRNDE stably transfected cells to generate miR-186+sh-CRNDE cells. Experiments with nude mice were conducted strictly in accordance with a protocol approved by the Administrative Panel on Laboratory Animal Care of the Shengjing Hospital. Four-week-old BALB/C athymic nude mice were purchased from the National Laboratory Animal Center (Beijing, China). The animals were free to autoclaved food and water during the study. The mice were divided into five groups randomly: control group (only GSCs), miR-186-NC group (miR-186-NC stable GSCs), miR-186 group (empty vector of miR-186 group), sh-CRNDE-NC group (empty vector of sh-CRNDE group), sh-CRNDE group (CRNDE inhibition stable GSCs), sh-CRNDE+miR-186 group (CRNDE inhibition and miR-186 overexpression stable GSCs). For subcutaneous implantation, 3 × 10^5^ cells were subcutaneously injected in the right flanks of the mice. Tumor volume was measured every five days when the tumors were apparently seen and calculated by the formula: volume (mm^3^) = length × width^2^/2. 28 days after implantation, mice were sacrificed and tumors were isolated.

### Statistical analysis

Experimental data were presented as means ± standard deviation (SD). Differences were analyzed by SPSS 18.0 statistical software with the Student's *t*-test or one-way ANOVA. Differences were considered significant if *P* < 0.05.
